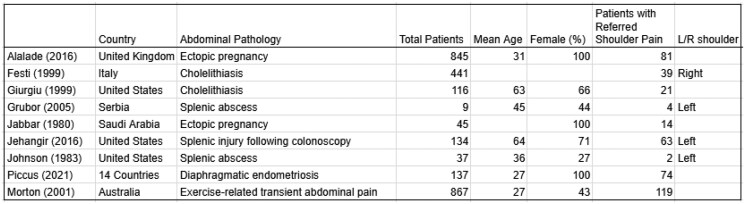# Poster Session I - A128 THE CLINICAL UTILITY OF REFERRED SHOULDER PAIN FOR THE DIAGNOSIS OF INTRA-ABDOMINAL PROCESSES: A SYSTEMATIC REVIEW

**DOI:** 10.1093/jcag/gwaf042.128

**Published:** 2026-02-13

**Authors:** D M Koerber, S Khan, M Byrne

**Affiliations:** Gastroenterology, The University of British Columbia, Vancouver, BC, Canada; Cambridge Memorial Hospital, Cambridge, ON, Canada; Gastroenterology, The University of British Columbia, Vancouver, BC, Canada

## Abstract

**Background:**

Referred shoulder pain is a phenomenon whereby non-musculoskeletal, noxious stimuli from outside of the shoulder leads to neurologic misinterpretation and perceived pain within the shoulder. This pain tends to originate from intra-abdominal sources, such as diaphragmatic irritation, and thus can serve as an important diagnostic clue in clinical practice. Despite its well-established physiological basis, referred shoulder pain is frequently overlooked or misattributed to musculoskeletal or orthopedic causes, delaying the diagnosis and management of potentially life-threatening abdominal processes. Further, little is known regarding the sensitivity or specificity of referred shoulder pain as a clinical tool.

**Aims:**

This review aims to evaluate existing literature to better understand the prevalence, mechanisms, and diagnostic relevance of this phenomenon, with the goal of improving early detection and management of intra-abdominal pathologies and minimizing unnecessary musculoskeletal diagnostic testing.

**Methods:**

The systematic review was conducted according to Preferred Reporting Items for Systematic Reviews and Meta Analyses (PRISMA) guidelines for scoping reviews using Medline, EMBASE, CINAHL, and Cochrane CENTRAL, with a systematic screening and extraction process. The search included articles published from database inception until December 2024 that reported incidence of referred shoulder pain in patients presenting with abdominal pathologies.

**Results:**

A total of 759 studies were eligible for title and abstract screening, and a total of 9 studies were ultimately included in the narrative synthesis. These consisted of prospective and retrospective cohort studies, cross-sectional studies, patient questionnaires, and case series. Collectively, the studies included 2,631 patients (mean age 42 years, 69% female). Referred shoulder pain was reported in 417 patients, corresponding to a pooled prevalence of 26%, with a range of 5 to 54%. Lateralization varied by etiology, with left-sided shoulder pain observed in splenic abscess and splenic injury from colonoscopy, right-sided in cholelithiasis, and variable presentation in ectopic pregnancy, diaphragmatic endometriosis, and exercise-related transient abdominal pain.

**Conclusions:**

Referred shoulder pain has significant clinical utility as a diagnostic marker for underlying intra-abdominal processes. This systematic review highlights how when present, referred shoulder pain can help to guide next steps in clinical diagnosis, particularly in the consideration of peri-diaphragmatic pathologies. Nevertheless, we noted that the sensitivity of referred shoulder pain was relatively low. Awareness of this phenomenon can enhance diagnostic accuracy, improve timely interventions, and ultimately lead to improved patient outcomes.

**Funding Agencies:**

None